# Herbivore Diet Breadth and Host Plant Defense Mediate the Tri-Trophic Effects of Plant Toxins on Multiple Coccinellid Predators

**DOI:** 10.1371/journal.pone.0155716

**Published:** 2016-05-16

**Authors:** Angelos Katsanis, Sergio Rasmann, Kailen A. Mooney

**Affiliations:** Department of Ecology and Evolutionary Biology, University of California Irvine, 321 Steinhaus Hall, Irvine, California, United States of America; Natural Resources Canada, CANADA

## Abstract

Host plant defenses are known to cascade up food chains to influence herbivores and their natural enemies, but how herbivore and predator traits and identity mediate such tri-trophic dynamics is largely unknown. We assessed the influence of plant defense on aphid and coccinellid performance in laboratory trials with low- vs. high-glucosinolate varieties of *Brassica napus*, a dietary specialist (*Brevicoryne brassicae*) and generalist (*Myzus persicae*) aphid, and five species of aphidophagous coccinellids. The performance of the specialist and generalist aphids was similar and unaffected by variation in plant defense. Aphid glucosinolate concentration and resistance to predators differed by aphid species and host plant defense, and these effects acted independently. With respect to aphid species, the dietary generalist aphid (vs. specialist) had 14% lower glucosinolate concentration and coccinellid predators ate three-fold more aphids. With respect to host plant variety, the high-glucosinolate plants (vs. low) increased aphid glucosinolate concentration by 21%, but had relatively weak effects on predation by coccinellids and these effects varied among coccinellid species. In turn, coccinellid performance was influenced by the interactive effects of plant defense and aphid species, as the cascading, indirect effect of plant defense was greater when feeding upon the specialist than generalist aphid. When feeding upon specialist aphids, low- (vs. high-) glucosinolate plants increased coccinellid mass gain by 78% and accelerated development by 14%. In contrast, when feeding upon generalist aphids, low- (vs. high-) glucosinolate plants increased coccinellid mass gain by only 11% and had no detectable effect on development time. These interactive effects of plant defense and aphid diet breadth on predator performance also varied among coccinellid species; the indirect negative effects of plant defenses on predator performance was consistent among the five predators when transmitted via the dietary specialist aphid, but these effects varied substantially among predators—in both the magnitude and direction—when transmitted via the dietary generalist aphid. Accordingly, the cascading effect of plant defense on predators was stronger in magnitude and more consistent among predator taxa when transmitted by the specialist than generalist herbivore. Overall, these findings support a central role of herbivore diet breadth in mediating both the strength and contingency of tri-trophic interactions.

## Introduction

Tri-trophic interactions have long been recognized as the key drivers of fundamental ecological and evolutionary processes [1–4]. At each trophic level, these interactions involve a multitude of different species or genotypes, and variation in the outcome of tri-trophic interactions is driven by diversity in the functional traits of the participating species [5, 6]. Accordingly, understanding how functional traits mediate the interactions among plants, herbivores and predators provides the mechanistic foundation for predicting the outcomes of cascading tri-trophic interactions in food chains [6, 7].

Plants display a combination of chemical and morphological defensive traits that reduce herbivory through both direct effects on herbivores, and indirectly, by mediating the top-down effects of natural enemies [8]. Such defensive traits are variable both within and among species [9] as a result of variable selection by herbivores [10], plant resource availability [11], and variable selection pressure of the predators [12]. Traits providing direct defense, by deterring or killing herbivores, include mechanical defenses and chemicals that act as poisons or digestibility reducers [8]. Traits providing indirect defenses serve to recruit natural enemies (predators or parasitoids) that consume or deter the herbivores [13, 14] through the production of rewards [15, 16] and cues that facilitate natural enemy location of herbivore prey [14, 17, 18]. Finally, direct and indirect defenses may act synergistically as, for example, when sub-lethal direct defenses slow herbivore development and thus increase susceptibility to natural enemies [19–23].

Herbivores in turn have their own offensive and defensive traits (including behaviors) that determine their ability to feed on plants and avoid natural enemies, respectively. Traits underlying herbivore offense include the tolerance, detoxification, deactivation or avoidance of plant defenses [24]. For example, herbivores may reduce plant chemical toxicity with detoxification enzymes [25] and deactivate (and thus avoid) mechanical defenses [26, 27]. Traits underlying herbivore defense against natural enemies include deterrent chemicals [28], behavioral, and morphological defenses [29]. However, herbivores may also use plants in their own defense against natural enemies, for example, when they manipulate or otherwise use plants for shelter [30] or sequester plant toxins to render themselves unpalatable [31, 32].

Herbivore diet breadth—the taxonomic or phenotypic diversity of plants consumed—is a key functional trait underlying much of the variation in herbivore offenses against plants and defenses against natural enemies [6, 33]. With respect to offense against plants, dietary specialist (mono- or oligophagous) herbivores often have physiological and behavioral adaptations that render them less sensitive to the plants’ direct defenses as compared to dietary generalists (polyphagous) herbivores that feed on a greater diversity of plants [34–36]. With respect to defense against natural enemies, many dietary specialists can sequester higher concentrations of plant toxins [33, 37] and may avoid natural enemies through superior unpalatability as compared to dietary generalists [6, 32].

Based upon the points outlined above, the tri-trophic interactions hypothesis [38] predicts that herbivore diet breadth plays a central role in mediating the bottom-up, cascading effects of plant defenses on both herbivores and natural enemies. With respect to plant-herbivore interactions, the performance of dietary generalist herbivores is predicted to be more sensitive to variation in plant defense than dietary specialists [37, 38]. Similarly, the tri-trophic interactions hypothesis predicts that variation in herbivore diet breadth should mediate the effects of plant defense on herbivore-natural enemy interactions [38]. For herbivores capable of sequestering plant toxins, the superior ability of dietary specialist herbivores to sequester [33, 37] means that variation in plant toxins should mediate predator effects more for dietary specialists than generalists. In support of this prediction, Francis et al. [39] and Jessie et al. [40] found that a specialist aphid was more toxic than a generalist aphid to coccinellid predators, and that such effects were stronger when aphids were reared on high- than low-glucosinolate plants.

In this study we tested the prediction that herbivore diet breadth mediates the effects of plant defenses on both plant-herbivore and herbivore-natural enemy interactions [38]. We studied two *Brassica napus* Linnaeus varieties of differing glucosinolate (the major secondary metabolite in Brassicaceae; [41, 42]) concentration, a dietary generalist (*Myzus persicae* Sultzer) and specialist (*Brevicoryne brassicae* Linnaeus) aphid species, and five species of coccinellid predators to address the following questions: How does herbivore diet breadth mediate the effects of plant defense on (1) aphid performance (i.e. plant resistance), (2) aphid resistance to coccinellids (i.e. the consumption rate of coccinellids = inverse of voracity) and (3) coccinellid performance (i.e. mass gain and developmental duration)? Furthermore, to assess the consistency of these dynamics, we compared our findings amongst five predators.

## Materials and Methods

### Plant and insect colonies

We took plants of differing toxicity and herbivores of differing diet breadths, and assessed the consequences of their interactions upon the performance of two aphid species and multiple species of predatory coccinellids. We selected two cultivars of canola, *Brassica napus* var. Amanda, a low glucosinolate cultivar, and *B*. *napus* var. Dwarf Essex, a high glucosinolate cultivar. These varieties were selected based on reported variation in glucosinolate concentration ([43]; JB Davis pers. comm.), and under the assumption that they should be similar with respect to other traits. Seeds of the two varieties were sown in 5 cm diameter pots containing an even mixture of sand, redwood bark, peat moss and perlite. After 1 week, the seedlings were transferred to the 10 cm diameter pots and grown under 23±1°C, 55% R.h., and 16 hrs daylight till the experimental manipulations.

The dietary generalist aphid *Myzus persicae* and the dietary specialist aphid *Brevicoryne brassicae* were chosen due to differing diet breadths and abilities to feed on the two Brassicaceae varieties. Both aphid species generally exhibit good performance and rapid population growth on both plant varieties tested in our study. *Myzus persicae* is highly polyphagous, capable of feeding on plants of at least 30 different plant families [44]. On the other hand, *B*. *brassicae* is oligophagous, limited to feeding on brassicaceous plants. Colonies of each aphid species were initiated from single individuals collected from *Brassica oleracea* L. plants in Orange County, California, USA. The two aphid species were inoculated on one-month-old plants and kept under the same light and temperature conditions as the plants.

Five coccinellid species were used in this study: *Anatis rathvoni* L., *Coccinella septempunctata* L., *Cycloneda sanguinea* L., *Harmonia axyridis* Pallas, and *Hippodamia convergens* L. All individuals used in experiments were F1 or F2 offspring from coccinellid adults collected from field sites, with permits from the University of California Natural Reserve System (UCNRS), in California, USA and specifically in Orange County (*C*. *septempuncta*, *C*. *sanguinea*, *H*. *axyridis*, *H*. *convergens*) and Mariposa County (*A*. *rathvoni*). With the exception of *A*. *rathvoni*, all coccinellid species tested live in a wide range of natural and agricultural habitats where *M*. *persicae B*. *brassicae*, can be found (S1 Table) [45, 46]. Finally, we also included *Anatis rathvoni*, majorly found in conifer forests, in order to increase the phylogenetic and ecological range of the studied species.

Beetles were reared following Katsanis et al. [47]. Specifically, coccinellid adults were housed in a 25°C glasshouse in 28 x 28 x 28 cm insect rearing cages (BugDorm, Taiwan) lined with filter paper. The cages contained low glucosinolate plants (*B*. *napus* var. Amanda) infested with high densities of the generalist aphid, *M*. *persicae*, a species palatable to most aphidophagous coccinellids [48]. Cages, filter paper and infested plants were changed each week. Coccinellid egg batches were carefully detached and placed in 9 cm Petri dishes lined with filter paper for eclosion. Neonate larvae were then used for the behavioral experiments described below, with the exception that a few were reserved for the continuation of the coccinellid culture.

### Aphid and plant traits

We measured several plant traits relating to growth and defense across the two plant varieties in order to assess potential variation besides chemical defenses (i.e. glucosinolates). All traits were measured on healthy, undamaged plants. After four weeks of growth, leaf toughness was measured by placing three leaves per plant between two plastic sheets with a 3 mm hole in the middle, and by measuring the force (measured in grams per surface area) needed to puncture a hole in the leaf using a penetrometer (PESOLA AG, Baar, Switzerland) (n = 12 plants per plant variety). Chlorophyll fluorescence, an indicator of photosynthetic activity, was measured with a chlorophyll meter (Konica Minolta Sensing Europe BV) over the leafy tissue of the same plants. Three measures were taken across three leaves per plants, and averaged. Specific leaf area (SLA) was determined by dividing the area of a 10 mm diameter leaf disc by the dry weight of the disc. Water content was measured by calculating the difference in weight between fresh and dry plant tissue. Finally, above ground plant biomass (i.e. growth rate) was measured by weighing dry leaf material. Leaves were dried at 60°C for 72 hrs. To measure leaf carbon (C), nitrogen (N) content and C:N ratios, dried leaves were ground to a fine powder using a Wig-L-bug grinding mill (International Crystal Laboratories, Garfield, NJ). Approximately 1 mg of this homogenized powder was then packed into 5 x 9 mm tins. Elemental analysis (NA 1500, Fisons Instruments, Ipswich, UK) was then performed at the UC Irvine Stable Isotope Ratio and Mass Spectrometry Facility (n = 6 plants per variety). For each trait, we tested for the effect of plant variety using t-tests in JMP v.10 (SAS Institute Inc., Cary, NC, USA).

Glucosinolate concentrations were assessed on both undamaged plants, plants damaged by each aphid species, and in the aphids themselves originating from the aphid performance experiment (see below). Leaves and 24 hr-starved aphids were dried at 50°C for 72 hours, weighed and ground to fine powder using a MM400 Retsch grinder (Retsch GmbH, Haan, Germany) in 2-mL Eppendorf tubes at 27 Hz for 2 min and 20 seconds, respectively. 20 nanograms of sinalbin was added to each sample as internal standard (CAS-No 20196-67-2, AppliChem GmbH, Darmstadt, Germany), and glucosinolates were extracted with boiling 70% methanolic solution, desulphatased with Sulfatase from *Helix pomatia* (CAS-No 9016-17-5, Sigma-Aldrich Co., St. Louis, IL, USA) on a DEAE-Sephadex A 25 column (CAS-No. 12609-80-2, Sigma) and separated on a C18 reversed phase LC column (Thermo Fisher Scientific Inc., Sunnyvale, CA, USA) on YL-9150 LC-PDA (YL Instruments Co. Ltd, Korea) with an acetonitrile water gradient as follows: 100–65% water and 0–35% acetonitrile over a period of 25 minutes followed by a final equilibration time of 5 minutes. Glucosinolates were identified using pure standards for most of the compounds and at 226 nm maximal absorbance spectra where the glucosinolates are characteristically peaking. Glucosinolate concentrations were calculated by dividing their areas with the area of the internal standard (sinalbin) and reported as micrograms per milligram dry tissue weight.

We tested for the effects of host plant variety (var. Amanda vs. var. Dwarf Essex), herbivory treatment (control, *M*. *persicae*, *B*. *brassicae*), and their interaction on plant glucosinolate concentrations with ANOVA. In addition, the main effect of glucosinolate compound identity (seven compounds, see below) was included in the model to account for variation in glucosinolate concentration among compounds. Tukey post-hoc tests were used to compare individual treatment levels or treatment combinations. Similarly, we conducted a parallel analysis testing for the effects of host plant variety, herbivore species and their interaction on aphid glucosinolate concentration, again including the main effect of glucosinolate compound identity (18 compounds, see below). In both analyses, glucosinolate data were log-transformed to meet assumptions of normality and analyses were again conducted using JMP v.10 (SAS Institute Inc., Cary, NC, USA).

### Aphid performance experiment

To measure the effect of host plant chemistry on aphid performance, 10 one-month old host plants from each plant variety were inoculated with one apterous adult from one of the two aphid species (i.e. 10 replicates for the generalist and 10 replicates for the specialist on each plant cultivar). After approximately 24 hours, the adult aphid and all but one neonate were then removed. These single neonate aphids of known age were then monitored each 24 hours to determine age at first reproduction and, subsequently for 5 days to determine rates of reproduction (number of nymphs produced). We assessed the effect of host plant variety, aphid species, and their interaction on age at first reproduction and fecundity with two-way ANOVAs using JMP v.10 (SAS Institute Inc., Cary, NC, USA). Data were log-transformed to meet assumptions of normally distributed residuals.

### Aphid resistance and coccinellid performance experiment

We here defined aphid resistance as the inverse of coccinellid predator voracity (i.e. the amount of aphids consumed by the coccinellid larvae) in order to assess treatment effects from the aphid’s perspective. In addition, the term ‘aphid resistance’ is as a direct analogy to the ‘plant resistance’ term sensu Karban and Baldwin [49], which reflects the amount of herbivore mortality due to predator feeding, in this case the coccinellid larvae.

Specifically, 10 newly hatched coccinellid larvae were individually put in 5 cm diameter Petri dishes. As aphid resistance is the integration of behavioral, morphological and chemical traits, by offering aphids to coccinellid larvae in a closed Petri dish arena, we aimed at minimizing the behavioral (escape) strategy, while accentuating the physico-chemical barriers to predator attack. For each larva, coccinellids were given live adult aphids, at a similar size, that had been feeding and developing for two weeks on four-week-old low- or high-glucosinolate plants at relatively low densities in order to avoid overcrowding and potential variation in aphid size due to competitive effects. Aphids were provided without any plant tissue to coccinellids at an abundance (“stocking level”) that was specific to each instar, and sufficient to exceed the daily rate of aphid consumption of the larvae: instar L1 = 5 aphids, L2 = 15, L3 = 30, L4 = 60. Each day the unconsumed aphids were counted, removed and replaced with fresh aphids at the appropriate stocking level. For each replicate, the experimental trial was ended one day after the coccinellid larvae had reached the 4^th^ instar. The fitness of a young adult coccinellid is tightly linked to the fitness of the 4^th^ larval instar [47], we thus considered unnecessary to continue the experiments into the adult stage.

Secondly, we measured coccinellid performance using two independent measures: 1) coccinellid larval mass gain and 2) larval developmental duration. Larval mass gain was calculated as the difference between the larval mass (in mg) at the beginning and end of each experimental trial. Developmental duration was calculated as the number of days required for a newly hatched coccinellid larva to reach the 4th instar. Once at the 4^th^ instar, larvae were given food for two additional days before the end of the experiment.

We assessed variation in aphid resistance, mass gain, and development duration using separate factorial three-way ANOVAs, with the main and interactive effects of plant variety, aphid species, and coccinellid species using JMP v.10 (SAS Institute Inc., Cary, NC, USA).

## Results

### Plant and aphid traits

As compared to the *B*. *napus* var. Dwarf Essex, var. Amanda had 18% greater biomass (S1A Fig, t_22_ = 2.96, p < 0.001) and leaves that were 38% tougher (S1B Fig, t_22_ = 4.86, p < 0.0001), 19% thicker (S1D Fig, t_10_ = -2.88, p = 0.009), but not different in C:N ratio (S1D Fig, t_10_ = 1.64, p = 0.133).

**Fig 1:**
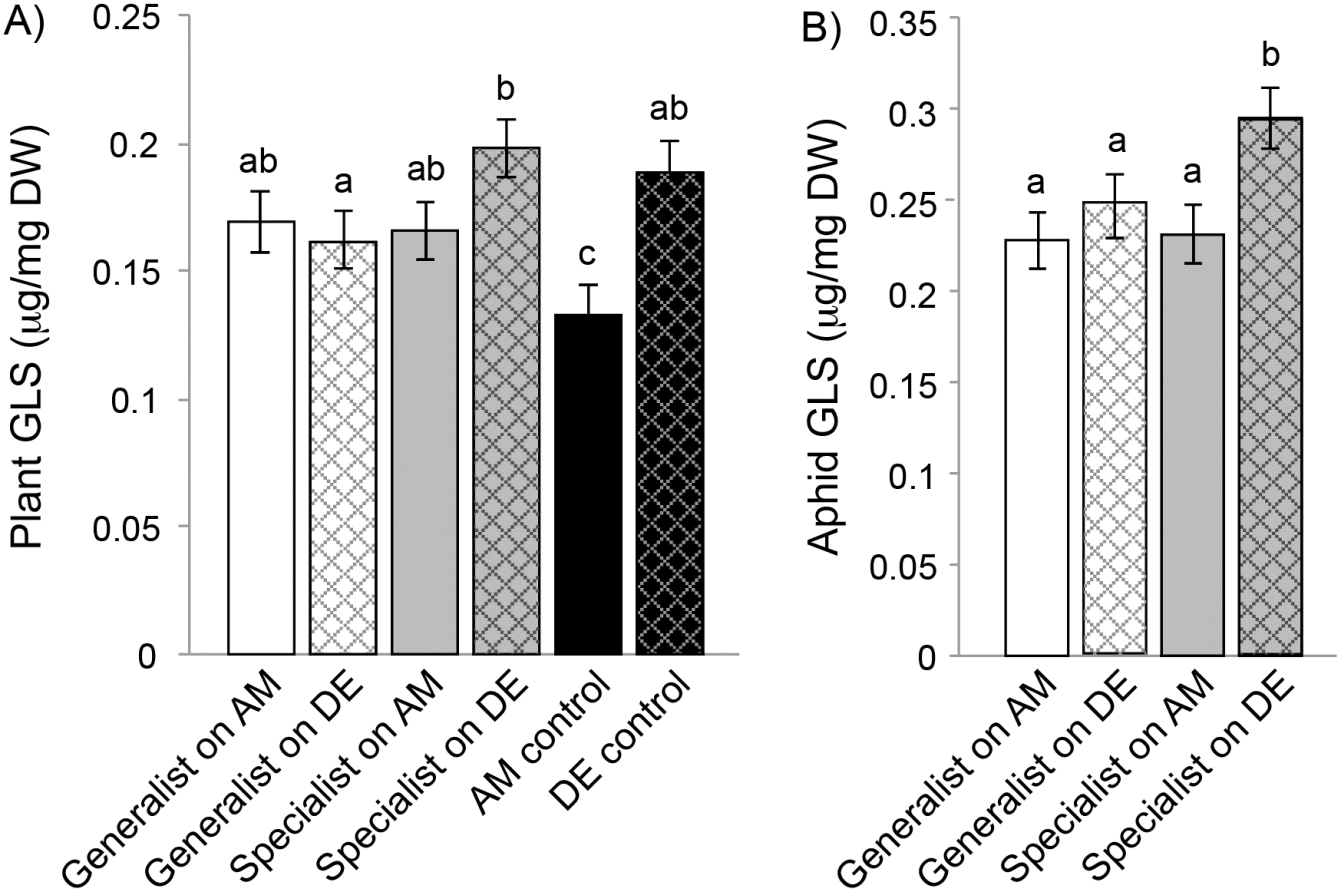
Glucosinolates (GLS) levels in A) leaves of two *Brassica napus* varieties following aphid herbivory. Means ± 1SE are shown for *Brassica napus* var. Dwarf Essex (DE) and var. Amanda (AM) after damage by *Brevicoryne brassicae* and *Myzus persicae* and for healthy (control) plants. Panel B) shown mean glucosinolate content in aphids’ bodies after feeding on both *B*. *napus* varieties. Letters above bars means significant differences (TukeyHSD test, p < 0.05).

We observed seven major glucosinolate compounds across the two varieties of *B*. *napus* (GLS 1, 3, 8, 9, 15, 16 and 17 as shown in Table 1). We found significant effects of plant variety, herbivory treatment, and a marginally significant variety-by-herbivory interaction (Table 2) on total glucosinolate abundance. On control (undamaged) plants, var. Dwarf Essex contained 30% higher total glucosinolate concentration than var. Amanda (Fig 1A). For var. Dwarf Essex, total glucosinolate concentration increased 5% by *B*. *brassicae* but decreased 14% by *M*. *persicae*. As a result, herbivore-induced Dwarf Essex had 24% higher total glucosinolate concentrations when fed upon by *B*. *brassicae* than *M*. *persicae*. For var. Amanda, total glucosinolate concentration increased to a similar extent by both *B*. *brassicae* and *M*. *persicae* (24% and 27% respectively) and, as a result, herbivore-damaged plants had similar glucosinolate concentrations regardless of aphid species.

**Table 1:** Total glucosinolate concentrations in plants and aphids.

GLS	AM/Mp		AM/Bb		DE/Mp		DE/Bb		AM/C	DE/C
	plant	aphid	plant	aphid	plant	aphid	plant	aphid	plant	
**1**	0.03	0.24	0.04	0.02	0.05	0.13	0.04	0.04	0.08	0.08
**2**	0	0.04	0	0.08	0	0.14	0	0.63	0	0
**3**	0.03	0.05	0.02	0.03	0.06	0.15	0.04	0.06	0.07	0.03
**4**	0	0.47	0	0.42	0	0.43	0	0.69	0	0
**5**	0	0.44	0	0.02	0	0.06	0	0.06	0	0
**6**	0	0.30	0	0.11	0	0.08	0	0.08	0	0
**7**	0	0.19	0	0.06	0	0.03	0	0.10	0	0
**8**	0.02	0.33	0.01	0.02	0.05	0.09	0.03	0.05	0.07	0.04
**9**	0.44	0.06	0.45	0.02	0.20	0.01	0.97	0.01	0.63	1.15
**10**	0	0.02	0	0.04	0	0.04	0	0.04	0	0
**11**	0	0.76	0	0.04	0	0.01	0	0.03	0	0
**12**	0	1.37	0	0.02	0	0.01	0	0.02	0	0
**13**	0	0.16	0	0.13	0	0.30	0	0.26	0	0
**14**	0	0.07	0	0.07	0	0.08	0	0.09	0	0
**15**	0.09	0.03	0.08	0.55	0.05	0.04	0.06	0.78	0	0.03
**16**	0	0.01	0	0.02	0	0	0	0.03	0	0
**17**	0.01	0.10	0.02	0.01	0.01	0.01	0.03	0.01	0	0.03
**18**	0.27	0.14	0.22	0.06	0.14	0.16	0.29	0.05	0	0.21
**Total**	0.89	4.78	0.84	1.72	0.54	1.77	1.46	3.03	0.85	1.57

Glucosinolates (GLS) concentrations in μg/mg of dry weight are shown for plant and aphid tissues for *Brassica napus* var. Amanda (AM) and var. Dwarf Essex (DE) after the attack of, *Myzus persicae* (Mp) (AM/Mp, DE/Mp) and *Brevicoryne brassicae* (Bb)(AM/Bb, DE/Bb) and in plant tissue from undamaged, control plants (AM/C, DE/C). Where ‘0’ is shown, glucosinolates were present in traces and thus not quantifiable. Numbered glucosinolates (in order of retention time) are Glucoraphanin (1), Sinalbin (2), and Glucobrassicin (5), with all others being unknown compounds.

**Table 2:** Results of statistical tests for the effect of plant variety and aphid herbivory on glucosinolate production and sequestration.

Dependent variable	Factor	*d*.*f*., error	F ratio	P value
**Plant GLS**	Plant variety	1, 231	5.42	.02
	Herbivory treatment	2, 231	1.32	.27
	P*H	2, 231	2.56	.08
	Compound identity	6, 231	79.97	<.001
**Aphid GLS**	Plant variety	1, 480	11.59	.001
	Herbivore species	2, 480	5.03	.02
	P*H	2, 480	0.31	.58
	Compound identity	17, 480	11.20	<.001

Two-way ANOVA summary table showing the main and interactive effects of plant variety (*Brassica napus* var. Amanda, *B*. *napus* var. Dwarf Essex), herbivory treatment (*Brevicoryne brassicae*, *Myzus persicae*, control, for plant GLS, and *B*. *brassica*, *M*. *persicae* for aphid GLS), and the main effect of glucosinolate compound identity.

We found a total of 18 major glucosinolate compounds in the two aphid species (Table 1). We found significant effects of aphid species, plant variety, but no aphid species-by-plant variety interaction on total glucosinolate abundance (Table 2). *Brevicoryne brassicae* contained 14% more glucosinolates than *M*. *persicae*, while aphids had 21% higher levels of glucosinolates when feeding on var. Dwarf Essex than var. Amanda (Fig 1B).

### Aphid performance

There was no significant effect of aphid species on age at first reproduction (F_1, 26_ = 0.53, p = 0.46; S2A Fig) or fecundity (F_1, 26_ = 0.01, p = 0.91; S2B Fig), and no effect of plant variety on age at first reproduction (F_1, 26_ = 0.01, p = 0.91) or fecundity (F_1, 26_ = 0.31, p = 0.59). Finally, the aphid*variety interaction was not significant for either age at first reproduction (F_1, 26_ = 0.21, p = 0.65) or fecundity (F_1, 26_ = 0.30, p = 0.59).

### Aphid resistance and coccinellid performance

**Fig 2:**
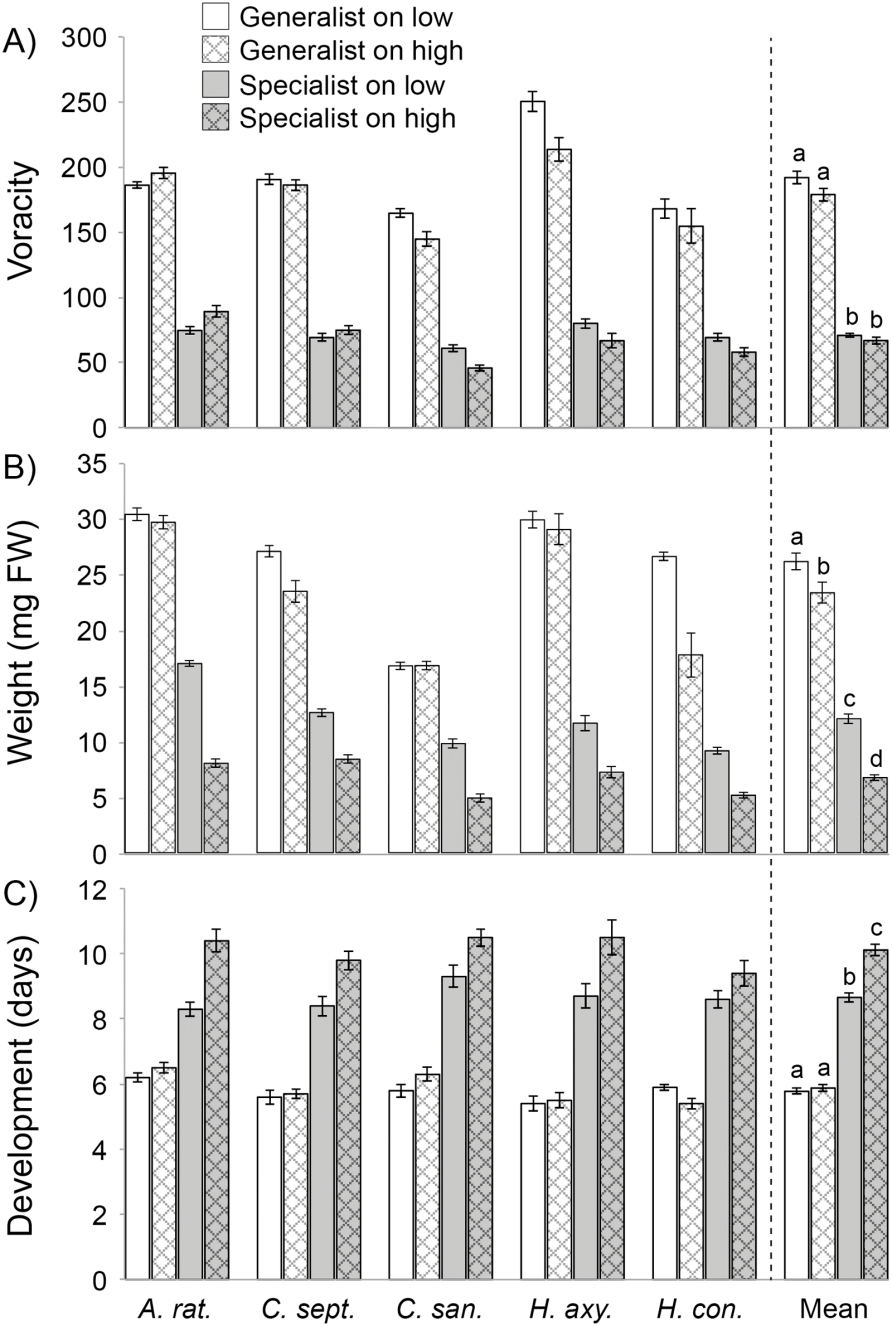
Effect of host plant chemical defense on aphid resistance and coccinellid performance. The panels show means ± 1SE for A) voracity (number of aphids consumed during development to the 4^th^ instar; inverse of aphid resistance), B) the development time in days to the 4th instar, and C) weight gain from to the 4th instars separately for 5 coccinellid species and overall means across all 5 species. The following coccinellid species were used: *Anatis rathvoni* (*A*. *rat*); *Coccinella septempunctata* (*C*. *sep*.), *Cycloneda sanguinea* (*C*. *san*.), *Harmonia axyridis* (*H*. *axy*.) and *Hippodamia convergens* (*H*. *con*.). Values are provided for *Brevicoryne brassicae* (black and dark gray bars) and *Myzus persicae* (light gray and open bars) feeding on either a low or high glucosinolate variety of *Brassica napus* (var. Amanda (AM) and var. Dwarf Essex (DE)). Letters above bars means significant differences (TukeyHSD test, p < 0.05).

Coccinellid voracity—and thus aphid resistance (the inverse of voracity)—was strongly influenced by both aphid species and plant variety, but there was no aphid species-by-plant variety interaction (Table 3, Fig 2A). Averaged across both plant varieties, coccinellids fed approximately three-fold more on *M*. *persicae* than on *B*. *brassicae*. Averaged across both aphid species, coccinellids fed 6% more on aphids coming from var. Amanda then var. Dwarf Essex. These effects of aphid species and plant variety differed among coccinellid species (aphid species-by-coccinellid species interaction, plant variety-by-coccinellid species interaction; Table 3). Voracity was consistently higher on *M*. *persicae* than *B*. *brassicae*, with variation among coccinellid species due to relatively small differences in the magnitude of this effect. In contrast, the indirect effects of host plant variety varied in both magnitude and direction of effect depending upon aphid and coccinellid species. When feeding upon *B*. *brassicae*, three coccinellid species (*Cycloneda sanguinea*, *H*. *axyridis*, *H*. *convergens*) had greater voracity for aphids from the low- than high-glucosinolate host plants (consistent with the mean effect across all species described above), while two species (*A*. *rathvoni*, *C*. *septempunctata*) expressed the opposing pattern. In contrast, when feeding upon *M*. *persicae*, four coccinellid species (*C*. *septempunctata*, *C*. *sanguinea*, *H*. *axyridis*, *H*. *convergens*) had greater voracity for aphids from the low- than high-glucosinolate host plants, while one species (*A*. *rathvoni)* expressed the opposing pattern.

**Table 3:** Results of statistical tests for the effect of plant variety and aphid herbivory on aphid resistance (inverse of coccinellid voracity) and coccinellid performance (larval mass gain and developmental duration).

Response variable	Factor	d.f., error	F ratio	P value
Voracity (-1*aphid resistance)	Coccinellid (C)	4,180	50.541	.000
	Herbivore (H)	1,180	2293.306	.000
	Plant (P)	1,180	12.142	.001
	C*H	4,180	20.36	.000
	C*P	4,180	7.34	.000
	H*P	1,180	3.48	.640
	C*H*P	4,180	0.613	.653
**Weight**	C	4,180	104.426	.000
	H	1,180	2254.218	.000
	P	1,180	155.902	.000
	C*H	4,180	29.639	.000
	C*P	4,180	5.190	.001
	H*P	1,180	14.818	.000
	C*H*P	4,180	11.661	.000
**Developmental duration**	C	4,180	4.395	.002
	H	1,180	832.572	.000
	P	1,180	39.968	.000
	C*H	4,180	2.634	.036
	C*P	4,180	1.997	.097
	H*P	1,180	30.377	.000
	C*H*P	4,180	0.618	.651

Three-way ANOVA table for the effects of five coccinellid species, two host plant genotypes, and the two aphid herbivore species, on the voracity (i.e. the inverse of aphid resistance), weight gain and developmental duration of coccinellid larvae.

Coccinellid larval weight gain was influenced by the interactive effects of aphid species and plant variety (Table 3, Fig 2B). Averaged across both plant varieties, coccinellids gained 2.6-fold more mass on *M*. *persicae* than on *B*. *brassicae*. Averaged across both aphid species, coccinellids gained 21% more mass on aphids coming from var. Amanda then var. Dwarf Essex. However, the plant variety effect was greater when coccinellids fed upon *B*. *brevicoryne* (78% more mass gain on var. Amanda then var. Dwarf Essex) than on *M*. *persicae* (11% more mass gain on var. Amanda then var. Dwarf Essex). These interactive effects of aphid species and plant variety in turn differed among coccinellid species (aphid species-by-plant variety-by-coccinellid species interaction; Table 3). When feeding upon *B*. *brassicae*, all coccinellid species had higher mass gain with aphids from the low- than high-glucosinolate host plants (consistent with the mean effect across all species described above). In contrast, when feeding upon *M*. *persicae*, only two coccinellid species (*C*. *septempunctata*, *H*. *convergens*) had higher mass gain with aphids from the low- than high-glucosinolate host plants, while two species were weakly affected (*A*. *rathvoni*, *H*. *axyridis*) and one species was unaffected (*C*. *sanguinea*) by host plant variety.

Mirroring the weight gain results, coccinellid developmental duration was influenced by the interactive effects of aphid species and plant variety (Table 3, Fig 2C). Averaged across both plant varieties, coccinellid development was 38% more rapid on *M*. *persicae* than on *B*. *brassicae*. Averaged across both aphid species, coccinellid development was 10% more rapid on aphids coming from var. Amanda then var. Dwarf Essex. However, the effect of plant variety was strong when coccinellids fed upon *B*. *brassicae* (14% more rapid development on var. Amanda then var. Dwarf Essex) but statistically undetectable when feeding upon *M*. *persicae* (Fig 2C). This interactive effect of aphid species and plant variety in turn differed among coccinellid species (aphid species-by-coccinellid species interaction, plant variety-by-coccinellid species interaction; Table 3). When feeding upon *B*. *brassicae*, all coccinellid species had more rapid development on aphids from the low- than high-glucosinolate host plants (consistent with the mean effect across all species described above). In contrast, when feeding upon *M*. *persicae*, three coccinellid species (*A*. *rathvoni*, *C*. *septempunctata*, *C*. *sanguinea*, *H*. *axyridis*) had more rapid development with aphids from the low- than high-glucosinolate host plants, while one species was weakly affected (*H*. *axyridis*) and one species (*H*. *convergens*) showed the reverse pattern.

## Discussion

### Summary

Herbivore performance and resistance to predators depended strongly on aphid diet breadth, with host plant variety being relatively unimportant. Aphid fecundity and age at first reproduction were unaffected by variation in host plant defenses (including glucosinolates) or other functional traits (including plant growth or leaf toughness), and did not differ with diet breadth (i.e. aphid species) (Table 1). However, there were independent and additive (non-interactive) effects of aphid diet breadth and host plant variety on both aphid glucosinolates and resistance to predators (Fig 2). Sequestration was 14% greater for the dietary specialist (*B*. *brassicae*) vs. the generalist (*M*. *persicae*) aphid (Fig 1B), and all five coccinellid predators ate dramatically more generalist than specialist aphids, resulting in a three-fold difference across predator taxa. In contrast, sequestration was 21% greater for aphids feeding upon the high- (var. Dwarf Essex) vs. low-glucosinolate (var. Amanda) glucosinolate host plants (Fig 1B), but coccinellid predators ate only 6% more aphids from low- than high-glucosinolate plants, and this effect varied in both magnitude and direction among coccinellid species. Accordingly, from the herbivore’s perspective, diet breadth mediated predator resistance but did not affect aphid performance in the absence of predators, while variation in host plant defense mediated aphid sequestration of glucosinolates but was inconsequential for both aphid performance and predator resistance.

In contrast to the effects on aphids, coccinellid performance was driven by the interactive effects of plant defense and aphid diet breadth. The cascading, indirect effect of plant defense on predator performance was greater when feeding upon the specialist than generalist aphid: When feeding upon specialists, low- (vs. high-) glucosinolate plants increased coccinellid mass gain 78% and accelerated development 14%. In contrast, when feeding upon generalists, low- (vs. high-) glucosinolate plants increased coccinellid mass gain by only 11% and had no detectable effect on development time. These interactive effects of plant defense and aphid diet breadth on predator performance in turn varied among coccinellid species; the indirect negative effects of plant defenses on predator performance were consistent among the five predators when transmitted via the dietary specialist aphid, but varied substantially among predators when transmitted via the dietary generalist aphid. Accordingly, the cascading effect of plant defense on predators was stronger in magnitude and more consistent among predator taxa when transmitted by the specialist than generalist herbivore. These findings support a central role of herbivore diet breadth in mediating both the strength and contingency of tri-trophic interactions.

### Herbivore performance and resistance in response to plant defense and herbivore diet breadth

Although the two aphid species studied differ dramatically in diet breadth, we did not observe the expected variation in performance or response to host plant defenses. The physiological efficiency hypothesis states that dietary specialists are better adapted than generalists at physiologically utilizing their host plants as food [34], and should thus have superior performance when feeding on their true host plant [35, 36], and that variation in host-plant defense should have stronger effects on dietary generalist than on better-adapted dietary specialist herbivores [37, 38]. In contrast to these predictions, we found that aphid performance (nymphs produced over a five-day period) was indistinguishable between the two species, despite the fact that *B*. *brassicae* feeds only on brassicaceous plants, while *M*. *persicae* feeds on at least 30 different plant families [44]. Furthermore, both aphids performed similarly on the two *B*. *napus* varieties, even though var. Dwarf Essex had 30% higher glucosinolate concentrations constitutively (control plants) than var. Amanda (Fig 1A). Accordingly, factors such as nutritional content of the plant, or other defensive metabolites such as non-protein amino acids, may be important for aphid resistance in this system [50].

In contrast, our findings for how plant defense and herbivore diet breadth mediate resistance to predators were more consistent with theoretical predictions. The enemy-free space hypothesis states that dietary specialist herbivores are better adapted than generalists at using their host plants for protection or defense from predators through their superior ability to sequester plant secondary compounds for their own defense [31, 33, 51, 52]. As predicted, and previously shown by Francis et al. [39], the dietary specialist aphid contained higher concentrations of glucosinolates than the dietary generalist (Fig 1B). Furthermore, variation in host plant defense also mediated variation in herbivore defense; both aphids contained somewhat higher concentrations of glucosinolates when feeding on the high- than low-glucosinolate plant variety, with 21% higher glucosinolates levels in aphids (across both species) being approximately proportional to the 30% difference in glucosinolates between the two host plants. The fact that this increase was similar in magnitude for both aphids shows that dietary specialization did not result in superior sequestration of plant defenses, in contrast with past studies [31, 33, 52, 53]. Furthermore, the equivalent shift in aphid and plant glucosinolate concentrations suggests that herbivore sequestration is constrained by the amount of secondary metabolite concentrations in the plants. Indeed, a linkage between plant secondary metabolites concentrations and insect sequestration has previously been shown with cardenolides in milkweeds (*Asclepias syriaca*) and monarch caterpillars (*Danaus plexippus*) [54] and with glucosinolates in aphids and brassicaceous plants [39], although sequestration is likely to be asymptotic when plant concentrations are very high [55].

Consistent with the enemy-free space hypothesis, the dietary generalist herbivore was consumed three-fold more than the dietary specialist [56]. Importantly, these strong differences in aphid resistance occur in the context of no difference in aphid performance in the absence of predators (Fig 1B). These findings thus suggest that the primary benefit of dietary specialization has more to do with predators than with plant-herbivore interactions [6, 57].

The dramatic differences in aphid resistance and palatability to predators were in turn notable given the relatively modest effects of diet breadth on aphid defense (i.e. glucosinolate concentration; Fig 1B). There are at least two potential and non-mutually exclusive mechanisms that could contribute to our results. First, the slightly higher concentrations of glucosinolates in the specialist aphid could stimulate relatively strong avoidance behaviors in predators [58]. To our knowledge, it is unknown whether coccinellid predators sense the toxicity of their aphid prey. However, there is evidence that some predators can detect herbivore-induced plant secondary metabolites [59, 60], and they may assess prey quality using volatile cues [39, 61]. Second, specialist aphids, but not the generalists, are able to simultaneously sequester not only glucosinolates, but also myrosinase enzymes [62]. Glucosinolate toxicity is only manifested when combined with myrosinases, as it is the degradation products of their interaction that produces biologically active compounds (i.e. isothiocyanates and nitriles; [63–65]). The ability of dietary specialists to sequester these enzymes may thus enhance their toxicity to predators. Accordingly, Francis [62] showed that the specialist *B*. *brassicae*, but not the generalist *M*. *persicae*, carried the myrosinase enzyme in its tissues, possibly explaining the greater resistance of *B*. *brassicae* to all five species of coccinellid larvae.

Although more modest in magnitude, we also observed effects of host plant defense on aphid resistance. Consistent with theoretical predictions [38] and past studies [37], variation in herbivore defense (Fig 1B) was in turn mirrored in resistance to predators, such that aphids feeding on high glucosinolate plants were less palatable to predators than aphids feeding on low glucosinolates plants (Fig 2A). This shows that a significant fraction of the variation in coccinellid developmental duration across a range of preys may be attributable to differences in their rate of consumption, rather than exclusively to differences in their dietary suitability. This is a factor that has not yet been considered in previous approaches that assessed dietary suitability in this group (e.g. [66]).

### Predator performance in response to plant defense and herbivore diet breadth

The dual effects of host plant defense and aphid diet breadth on aphid resistance strongly affected the performance of all five coccinellid species. Several decades of research has shown that sequestration of plant secondary metabolites by insect herbivores can affect the development, growth and fecundity of predators [2, 32, 67, 68]. Here we show that both increased plant defense and a more specialized herbivore diet breadth reduce predator performance. Although these two effects operated independently with respect to aphid resistance, their effects were not independent from the point of view of predator performance; the negative indirect effects of host plant defense were stronger when transmitted via the dietary specialist than generalist aphid. These interactive effects were especially strong with respect to predator development time, whereas there was no indirect effect of host plant defense transmitted via the dietary generalist, but a development time with the dietary specialist was 17% longer when the aphid had fed upon the high than low glucosinolate plant variety.

The cascading effects of host plant defense on predator performance were consistent when transmitted via the dietary specialist aphid, but varied substantially among the five coccinellid species—in both magnitude and direction—when feeding upon the dietary generalist aphid. As described above, the high toxicity of the dietary specialist herbivore could be the result of the combined sequestration of the glucosinolate compounds and the myrosinase enzymes. The homogenous, negative response of coccinellids to host plant defense (i.e. 78% more mass gain and 14% more rapid development on low-glucosinolate plants) thus suggests phylogenetically conserved (and perhaps even constrained) mechanisms of glucosinolate detoxification in these predators. In contrast, if the dietary generalist aphid does not sequester myrosinase enzymes, resistance may be based upon traits other than glucosinolate sequestration, with the efficacy of these traits in turn being contingent upon predator identity. This variation in predator response appears to be unrelated to the habitat preference (S1 Table), but may be driven by other unknown characteristics.

## Conclusions

There is a growing recognition for the importance of a tri-trophic perspective in plant-herbivore and herbivore-predator interactions, and of the insufficiency of studying pairwise interactions in a community-level context [6, 18, 38, 57, 69]. Our findings in turn demonstrate the central role played by herbivore diet breadth, as the cascading effect of plant defense on predators was stronger in magnitude and more consistent among predator taxa when transmitted by the specialist than generalist herbivore.

## Supporting Information

S1 Fig Plant traits. Shown are the averages (+/- 1SE) of A) plant dry biomass, B) leaf toughness measured as the force needed to pierce a 3 mm diameter hole punch in each leaf, C) specific leaf area (SLA), and D) carbon to nitrogen ration (C/N) for *Brassica napus* variety Amanda (AM), and *B*. *napus* variety Dwarf Essex (DE). Asterisks between bars means significant difference across *B*. *napus* varieties (t-test, p < 0.05).
(TIF)

S2 Fig Effect of host plant chemical defense on aphid performance. The panels show means ± 1SE for A) age at first reproduction and, B) number of offspring. Values are provided for *Brevicoryne brassicae* (black and dark gray bars) and *Myzus persicae* (light gray and open bars) feeding on either a low or high glucosinolate variety of Brassica napus (var. Amanda (AM) and var. Dwarf Essex (DE)). Letters above bars means significant differences (TukeyHSD test, p < 0.05).
(TIF)

S1 Table Relative size index (pers. observation) and habitat for the five coccinellid species tested. Species abbreviations are mentioned in Fig 2.
(DOCX)
