# Supplementary material for: Herbivore Diet Breadth and Host Plant Defense Mediate the Tri-Trophic Effects of Plant Toxins on Multiple Coccinellid Predators
Source: PLoS One. 2016 May 16;11(5):e0155716. doi: 10.1371/journal.pone.0155716 (PMC4868332; doi:10.1371/journal.pone.0155716)
Supplement: S1 Table — Species abbreviations are mentioned in Fig 2. (DOCX) [file pone.0155716.s003.docx]

| **Relative size** | **Species** | **Habitat** |
| --- | --- | --- |
| 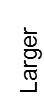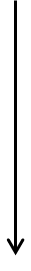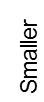 | *A. rat.* | conifers |
|  | *H. axy.* | conifers, semi-arboreal, meadows, agricultural |
|  | *C. sept.* | conifers, semi-arboreal, meadows, agricultural |
|  | *H. con.* | semi-arboreal, meadows, agricultural |
|  | *C. san.* | semi-arboreal, agricultural |
